# Lassa Virus in Pygmy Mice, Benin, 2016–2017

**DOI:** 10.3201/eid2510.180523

**Published:** 2019-10

**Authors:** Anges Yadouleton, Achaz Agolinou, Fodé Kourouma, Raoul Saizonou, Meike Pahlmann, Sonia Kossou Bedié, Honoré Bankolé, Beate Becker-Ziaja, Fernand Gbaguidi, Anke Thielebein, N’Faly Magassouba, Sophie Duraffour, Jean-Pierre Baptiste, Stephan Günther, Elisabeth Fichet-Calvet

**Affiliations:** Laboratoire des Fièvres Hémorragiques Virales, Cotonou, Benin (A. Yadouleton, A. Agolinou);; Ministry of Health, Cotonou (A. Yadouleton, H. Bankolé, F. Gbaguidi);; Laboratoire des Fièvres Hémorragiques Virales, Conakry, Guinea (F. Kourouma, N. Magassouba);; World Health Organization, Cotonou (R. Saizonou, S.K. Bedié, J.-P. Baptiste);; Bernhard-Nocht Institute for Tropical Medicine, Hamburg, Germany (M. Pahlmann, B. Becker-Ziaja, A. Thielebein, S. Duraffour, S. Günther, E. Fichet-Calvet)

## Abstract

Lassa virus has been identified in 3 pygmy mice, *Mus baoulei*, in central Benin. The glycoprotein and nucleoprotein sequences cluster with the Togo strain. These mice may be a new reservoir for Lassa virus in Ghana, Togo, and Benin.

Lassa fever has recently emerged in Benin and Togo, where it had been unknown until 2014. In November 2014, two persons died of confirmed Lassa virus (LASV) infection at Saint Jean de Dieu Hospital, in Tanguieta, northern Benin. During January–February 2016, a second outbreak with 11 confirmed cases of Lassa fever occurred in the communes of Tchaourou and Parakou, department of Borgou, central Benin. These 11 cases were diagnosed at the Irrua Specialist Teaching Hospital (Irrua, Nigeria) and the Bernhard-Nocht Institute for Tropical Medicine (Hamburg, Germany). During the same period, 2 cases from neighboring Togo were also confirmed as Lassa fever (*1*,*2*).

In July 2016, to enable the affected countries to quickly detect new cases of Lassa fever, the Bernhard-Nocht Institute for Tropical Medicine and the German Ministry of Cooperation established LASV diagnostic capacity in Cotonou (Benin) and Lomé (Togo). In 2017, another 2 cases occurred in central Benin. 

The need to understand the epidemiology of Lassa fever in Benin and the involvement of rodents in the transmission of the disease led us to investigate the small mammal community living in and around the dwellings in villages where the index case-patients lived. To identify these villages, a first expedition in October 2016 traced back confirmed and probable cases according to the health registers of the local hospital in Tchaourou and the teaching hospital in Parakou. An investigation of several villages enabled us to record some evidence from the nurses in the health centers.

On the basis of these findings, a second expedition in September 2017 used Sherman traps (https://www.shermantraps.com) to capture small mammals in 6 villages in Tchaourou. The animals were sampled in several habitats: houses (inside, 80 traps) and fields and savannah (outside, 120 traps). The animals were then killed with an overdose of halothane, and necropsies were performed in situ according to Biosafety Level 3 security procedures (*3*).

We collected blood and organs (including spleen and liver) and identified the animals morphologically, according to standard measurements: body weight; body, tail, hindfoot, and ear lengths. Because of possible sibling species among *Mastomys* spp. and *Mus* spp. rodents, we performed molecular identification through a PCR targeting cytochrome b. Distribution of the small mammals was 210 *Praomys daltoni* mice, 14 *Mus baoulei* mice, 12 *Rattus rattus* rats, 10 *Lemniscomys striatus* mice, 7 *Mus mattheyi* mice, 6 *Mastomys natalensis* mice, and 26 *Crocidura* spp. shrews (Appendix Table 1). The surprising finding was the scarcity of *M. natalensis* mice, the most probable reservoir of LASV; we trapped only 3 of these mice inside and 3 outside. In that area, the commensal rodent was *P. daltoni*, as is often found in Ghana and Nigeria (*4*,*5*).

We screened all samples for LASV by using 2 reverse transcription PCRs: 1 specific for LASV and 1 for panarenaviruses (*6*,*7*). The 2 tests enabled us to detect 3 LASV-positive animals, all pygmy mice (*M. baoulei*). To determine phylogeny more reliably than we could by using short fragments issued from the diagnostic tests, we performed additional PCRs on glycoprotein (GP) and nucleoprotein (NP) genes located on the small RNA segment (primers in Appendix Table 2). GP sequence of 1,408 nt and NP sequences of 1,654 nt were aligned with 31 LASV sequences belonging to all lineages.

The phylogenetic analyses performed with a Bayesian approach on GP and NP alignments shows that the 3 new sequences (Worogui50, Worogui51, and Odo-Akaba13) clustered with Jirandogo76, from the same species (*M. baoulie*) collected in Ghana in 2011 (Figure). Furthermore, the analysis showed strong support with the strains from humans in Togo, which clustered with the sequences from humans in Benin (S. Günther, E. Fichet-Calvet, unpub. data). The differences between the 3 GP sequences in mice from Benin and the strain from humans in Togo ranged from 20.8% to 21.7% (8.5% to 10.3% at the amino acid level). Differences between NP sequences ranged from 22.2% to 25.7% (10.2% to 13.6% at the amino acid level). Jirandogo76 is closer to Togo on the NP tree, with a difference of 21% nt (8.9% at the amino acid level). These findings are consistent with past observations highlighting the high amino acid variability among LASV strains (*9*). This finding suggests that the sequences described for *M. baoulei* mice belong to the Lassa clade. Nevertheless, the node dividing the branches of the Togo strain from those of the newly identified LASV in *M. baoulei* mice is deep, suggesting that these strains diverged a long time ago by switching hosts between rodents and humans. Additional reservoirs could still be implicated in the recent events of LASV transmission to humans. 

**Figure:**
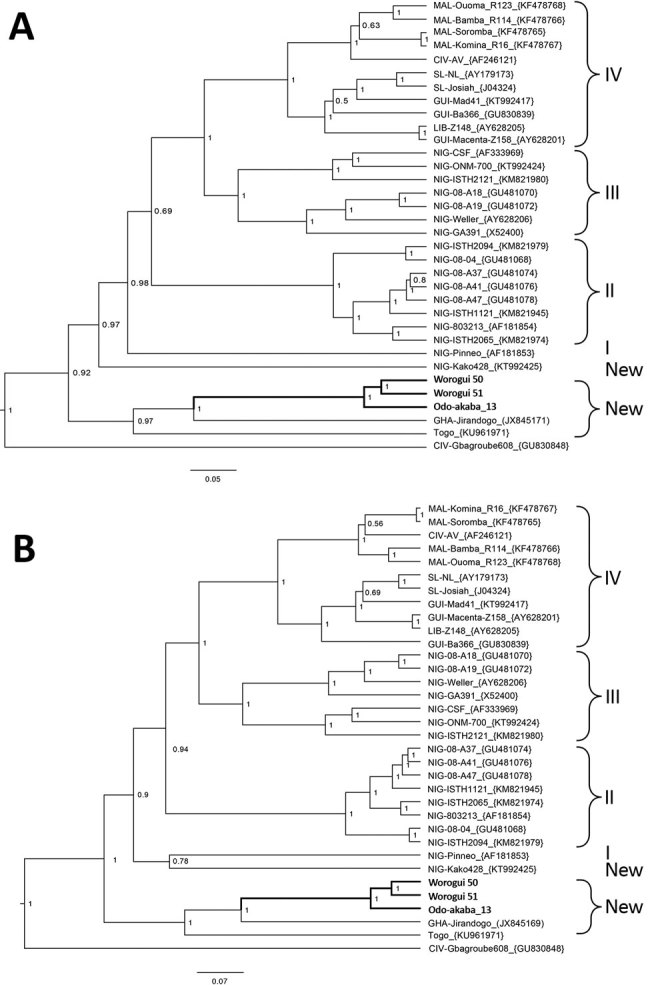
Bayesian phylogenetic analyses based on nucleotide sequences of the partial glycoprotein and nucleoprotein genes of Lassa virus (LASV), showing the placement of the new sequences (in boldface) isolated from *Mus baoulei* pygmy mice, in comparison with other sequences representing the members of LASV lineages I–IV. A) Glycoprotein, 1,408 nt; B) nucleoprotein, 1,654 nt. The trees are rooted by Gbagroube, a LASV-like virus isolated from *Mus setulosus* mice in Côte d’Ivoire. Statistical support of grouping from Bayesian posterior probabilities is indicated at the nodes. Country, strain names, and GenBank accession numbers are indicated on the branches. The analysis was inferred by using the Bayesian Markov chain Monte Carlo method implemented in BEAST (*8*). The following settings were used: general time reversible plus gamma, strict clock, and constant population. Markov chain Monte Carlo chains were run for 10 million states and sampled every 10,000 states to obtain an effective sample size >200 for all parameters. The new viral and murine sequences are deposited under accession nos. MH028396–404. Scale bars indicate nucleotide substitutions per site.

Our findings strongly point toward *M. baoulei* mice as a potential candidate for LASV spreading in Benin, Togo, and Ghana. Together with the multimammate mice *M. natalensis* and *Mastomys erythroleucus* and the soft-furred mouse *Hylomyscus pamfi* (*10*), the fourth rodent species reservoir of LASV is *M. baoulei* pygmy mice.

Appendix Small mammals captured and primers used in study of Lassa virus in pygmy mice, Benin, West Africa, 2016–2017.
