# Supplementary material for: Lassa Virus in Pygmy Mice, Benin, 2016–2017
Source: Emerg Infect Dis. 2019 Oct;25(10):1977–9. doi: 10.3201/eid2510.180523 (PMC6759236; doi:10.3201/eid2510.180523)
Supplement: Appendix — Small mammals captured and primers used in study of Lassa virus in pygmy mice, Benin, West Africa, 2016–2017. [file 18-0523-Techapp-s1.pdf]

Article DOI: https://doi.org/10.3201/eid2510.180523

Lassa Virus in Pygmy Mice, Benin, West Africa, 2016–2017

Appendix

Appendix Table 1. Small mammals captured in 6 villages in Tchaourou commune, Benin, West Africa*

Species	Worogui	Kassouala	Kadjola	Yambouan	Gango	Odo-Akaba	Total
Crocidura spp	1/6	1/4	0/2	0/3	1/4	1/3	26
Lemniscomys striatus	0/3			0/3		0/4	10
Mastomys natalensis	0/3			3/0			6
Mus (*Nannomys*) *baoulei*	0/2					0/12	14
Mus (*Nannomys*) *mattheyi*	1/2	0/1		2/1			7
Praomys daltoni	41/0	62/4	29/6	3/2	41/2	20/0	210
Rattus rattus	0/1	3/0	1/0	2/0	0/1	4/0	12
Total	60	75	38	19	49	44	285

*Numbers of captures are presented by habitat (inside/outside). Worogui: 8°53′2.21”N, 2°40′18.57”E, Kassouala: 8°52′28.78”N, 2°45′9.88”E, Kadjola: 8°54′44.07”N, 2°43′11.95”E, Yambouan: 8°58′25.84”N, 2°45′2.68”E, Gango: 8°58′18.90”N, 2°41′47.27”E, Odo-akaba: 8°46′24.07”N, 2°36′10.29”E.

Appendix Table 2. Primers used in study of Lassa Virus in pygmy mice, Benin, West Africa, 2016–2017

Label	Oligosequence, 5’→3’	Target gene	Reference
LVS36+	ACC GGG GAT CCT AGG CAT TT	GP	(1)
LVS 339-	GTT CTT TGT GCA GGA MAG GGG CAT KGT CAT	GP	(*1*)
LVS 732+	CCARAACACCACCTGGGAAGATCAYTG	GP	(*2*)
OWS 1000-	AGCATGTCACAGAAYTCYTCATCATG	GP	(3)
LVS 1474-	ATGCCCATGTGRTTSAGYCTRTG	GP	(*2*)
LVS 1607+	GGTGTTGATGTTCTAAASACC	NP	(*4*)
LVS 1673+	CCCGACACTGCTGCATCAAACATG	NP	this study
OWS 2120+	GGTCTCCCTTCAATGTCMATCCA	NP	(3)
LVSnig 2511-	TGTTGGAGACCATCAAGGTT	NP	this study
LVSnig 2541-	CTGGAGCCTGTATGCTTGAT	NP	this study
LVS 2656a+	GTTGGGGTACTTTGCTGTGTA	NP	this study
OWS 2840b-	AAYAAYCAGTTTGGGACNATGCC	NP	(*3*)
OWS 3400-	GCGCACAGTGGATCCTAGGC	NP	(3)
LVL 3359D Y+	AGAATCAGTGAAAGGGAAAGCAAYTC	L	(*5*)
LVL 3359G Y+	AGAATTAGTGAAAGGGAGAGTAAYTC	L	(5)
LVL 3754A R-	CACATCATTGGTCCCCATTTACTATGRTC	L	(5)
LVL 3754D R-	CACATCATTGGTCCCCATTTACTGTGRTC	L	(5)
L7	ACC AAT GAC ATG AAA AAT CAT CGT T	Cytochrome b	(6)
H15915	TCT CCA TTT CTG GTT TAC AAG AC	Cytochrome b	(6)
F-49	CAT TCA TTG ACC TAC CTG CT	Cytochrome b	(7)
R-505	AGA ATC CCC CTC AAA TTC AC	Cytochrome b	(7)
F-607	CGG GCT CTA ATA ACC CAA CG	Cytochrome b	(7)
R-813	TTC TGG TTT GAT ATG GGG AGG T	Cytochrome b	(7)

*GP, glycoprotein; L, large RNA segment; NP, nucleoprotein.

References

1. Olschläger S, Lelke M, Emmerich P, Panning M, Drosten C, Hass M, et al. Improved detection of Lassa virus by reverse transcription-PCR targeting the 5′ region of S RNA. J Clin Microbiol. 2010;48:2009–13. PubMedhttps://doi.org/10.1128/JCM.02351-09

2. Olayemi A, Cadar D, Magassouba N, Obadare A, Kourouma F, Oyeyiola A, et al. New Hosts of The Lassa Virus. Sci Rep. 2016;6:25280. PubMedhttps://doi.org/10.1038/srep25280

3. Ehichioya DU, Hass M, Becker-Ziaja B, Ehimuan J, Asogun DA, Fichet-Calvet E, et al. Current molecular epidemiology of Lassa virus in Nigeria. J Clin Microbiol. 2011;49:1157–61. PubMedhttps://doi.org/10.1128/JCM.01891-10

4. Fichet-Calvet E, Ölschläger S, Strecker T, Koivogui L, Becker-Ziaja B, Camara AB, et al. Spatial and temporal evolution of Lassa virus in the natural host population in Upper Guinea. Sci Rep. 2016;6:21977. PubMedhttps://doi.org/10.1038/srep21977

5. Vieth S, Drosten C, Lenz O, Vincent M, Omilabu S, Hass M, et al. RT-PCR assay for detection of Lassa virus and related Old World arenaviruses targeting the L gene. Trans R Soc Trop Med Hyg. 2007;101:1253–64. PubMedhttps://doi.org/10.1016/j.trstmh.2005.03.018

6. Ducroz JF, Granjon L, Chevret P, Duplantier JM, Lombard M, Volobouev V. Characterization of two distinct species of *Arvicanthis* (Rodentia: Muridae) in West Africa: cytogenetic, molecular and reproductive evidence. J Zool (Lond). 1997;241:709–23. https://doi.org/10.1111/j.1469-7998.1997.tb05743.x

7. Lecompte E, Brouat C, Duplantier JM, Galan M, Granjon L, Loiseau A, et al. Molecular identification of four cryptic species of *Mastomys* (Rodentia, Murinae). Biochem Syst Ecol. 2005;33:681–9. https://doi.org/10.1016/j.bse.2004.12.015
